# Crescent Fracture of the Pelvis: A Case Report

**DOI:** 10.7759/cureus.32944

**Published:** 2022-12-25

**Authors:** Ariba Ahmed, Aditya L Kekatpure, Aashay Kekatpure

**Affiliations:** 1 Orthopaedics, Jawaharlal Nehru Medical College, Datta Meghe Institute of Higher Education and Research, Wardha, IND; 2 Orthopaedic Surgery, Jawaharlal Nehru Medical College, Datta Meghe Institute of Higher Education and Research, Wardha, IND; 3 Orthopaedic Surgery, NKP Salve Medical College and Hospital, Nagpur, IND

**Keywords:** dislocation, outcome, injuries, operative, lateral compression, pelvis, crescent fracture

## Abstract

Crescent fractures are a rare type of pelvic injury. They result from a lateral compression force and are rotationally unstable. The following is a case of a young male who suffered a crescent fracture on the right side of the pelvis and was treated for the same. These fractures, being rare and complex, need to be managed in accordance with other injuries sustained by the patient and also need fixation for a better functional outcome.

## Introduction

After the groundbreaking work of Judet and Letournel in the early 1960s, pelvic and acetabular surgery underwent a slow progression. Although the surgical techniques have steadily grown less intrusive, the target of attaining structural and functional stabilization of the acetabulum and nearly anatomical repair of the pelvic ring has remained the same. The treatment of choice for acetabular fractures involving the weight-bearing dome or with displacement greater than 2 mm in the last two decades has been open reduction and internal fixation (ORIF), with reports of positive outcomes.

Due to the conglomerate structure of the intra-pelvic tissues, internal fixation has always been a challenging procedure, and therefore the best surgical course of action for pelvic ring injuries is still a matter of debate. The pelvic ring is most frequently damaged by high-energy trauma. Hemorrhage and associated thoracic and brain damage account for 10% to 20% of all fatalities. Conventionally, the internal fixation approach has always necessitated considerable surgical exposure of the deep pelvic tissues, which raises the risk of infection by 25% and may delay wound healing or injure major arteries or nerves. These side effects are primarily a result of the surgical management itself rather than the main injury. It seems logical to consider less invasive methods as a result. Percutaneous fixation of the pelvis has received a lot of attention in this effort to reduce the morbidity of such invasive surgical techniques and approaches. For sacroiliac dislocation and sacral fractures, percutaneous fixation of the pelvis was initially addressed by Routt et al. [1]. He successfully demonstrated it to be a safe and efficient interventional approach, which is bio-mechanically stable with significantly less rates of bleeding and infection. Consequently, the use of iliosacral screws for intra-cutaneous fixation has become more widespread for treating complex pelvic ring injuries. Closed reduction with percutaneous fixation of non-displaced or minimally displaced fractures of the acetabulum under CT or fluoroscopic guidance has attracted interest due to recent developments in imaging and computer navigation techniques [2].

Fractures occurring due to a laterally compressing force account for nearly 80% of all injuries occurring to the pelvic ring. Despite the fact that it is impossible to determine the exact percentage from the literature, crescent fractures are a less common subgroup of this category. A well-known subtype of pelvic ring injuries caused by lateral compression stress is crescent fracture-dislocations. They are characteristically identified by sacroiliac joint dislocation, which extends as a fracture of the posterior iliac ring. They are essentially sacroiliac joint fracture-dislocations, with varying degrees of sacroiliac ligament complex rupture and an anteriorly expanding fracture of the posterior iliac crest. The unharmed portion of the posterior ligament complex keeps a half-moon-shaped portion of the iliac wing anchored to the sacrum. The typical crescent fracture topography can be seen on the anteroposterior (AP) radiograph.

Patients with posterior pelvic ring injuries typically have pubic rami fractures or symphyseal diastasis. Since fatal pelvic vascular injuries are not frequently associated with crescent fractures, an external pelvic fixator device in compression is typically not needed right away. Urogenital and vascular injuries may worsen any pelvic ring fracture or dislocation, thus they should not be ignored. It is now more straightforward to select patients who will benefit from angiography and vascular embolization operations thanks to contrast-enhanced CT scans. Surgical management is suggested for the reduction and stabilization of a crescent fracture-dislocation. The incidence of mal-union, post-traumatic arthritis of the sacroiliac joint, uncomfortable stance phase gait cycle instability, and seated obliquity should decrease with the restoration of the normal architecture. Some patients may endure prolonged pain and dysfunction despite adequate reduction and stabilization, which is frequently seen in high-energy injuries [3]. Day et al. categorizes crescent fractures as follows: type I fractures enter the joint inferiorly and affect less than one-third of it. The fracture line enters the joint not far from the anterior S2 nerve root foramen, which is best seen in three-dimensional (3D) CT datasets and outlet views of the pelvis. A sizable crescent-shaped fragment is also visible. Through the use of an ilioinguinal technique, this fracture type may be surgically treated. Almost all the time, the lateral window provides adequate exposure and makes it easier to use an anterior plating approach with direct visibility of the fracture and sacroiliac joint. In type II fractures, the joint is affected by one-third to two-thirds of the fracture. The line of discontinuity enters the junction between the anterior foramen of S1 and S2 on the moderately sized crescent fragment. This is the fracture type that Borrelli et al. documented and it can be surgically treated using a posterior technique that makes it easier to insert inter-fragmentary screws and supplemental plates. These lateral compression injuries, according to Borrelli et al., may be linked to serious soft-tissue abrasions like the Morel-Lavallee lesion [4,5]. Type III fractures have a tiny, superior crescent fragment and affect more than two-thirds of the joint. The anterior S1 nerve root foramen is where the crack enters the joint from the back and above. To treat this fracture type medically, a closed or percutaneous reduction approach may be used, along with the implantation of percutaneous iliosacral screws. Only well-trained practitioners should use the procedure, and a closed reduction might not be feasible in cases of delayed presentation. Given these facts, it could be claimed that the lateral window of an ilioinguinal approach provides the best opportunity for precise reduction and secure anterior plate attachment [6,7].

## Case presentation

History

A young adult male patient aged 21 years presented to the emergency department with a history of automobile collision with a run-over injury by a four-wheeler. In his initial assessment, he was hemodynamically stable and no visceral, cerebral, or secondary injuries were diagnosed. After an initial primary survey to rule out other associated injuries, a pelvis X-ray with both hips was done, which revealed pelvic ring injury involving both anterior and posterior pelvic rings. The patient did not have any chronic or congenital disorder, which was also confirmed later by several tests.

Preoperative planning

**Figure 1:**
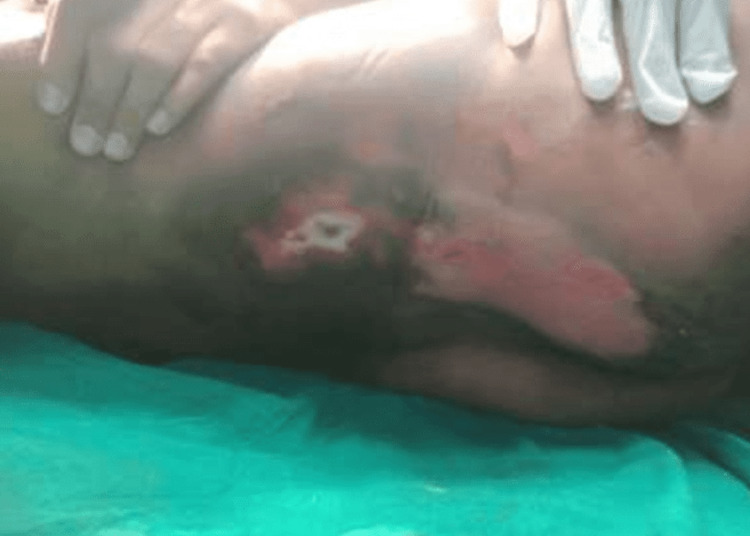
Image showing the externally visible soft tissue injury sustained by the patient

**Figure 2:**
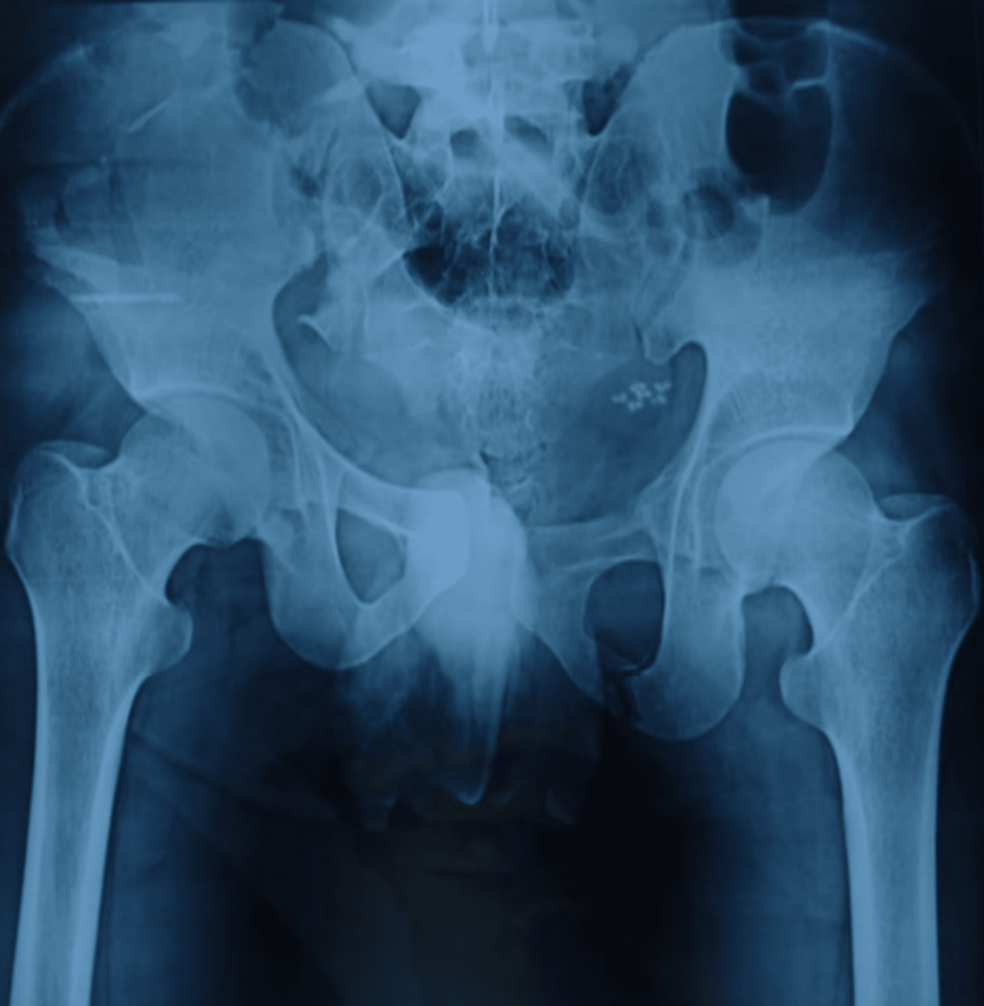
Pre-operative X-ray of the patient

**Figure 3:**
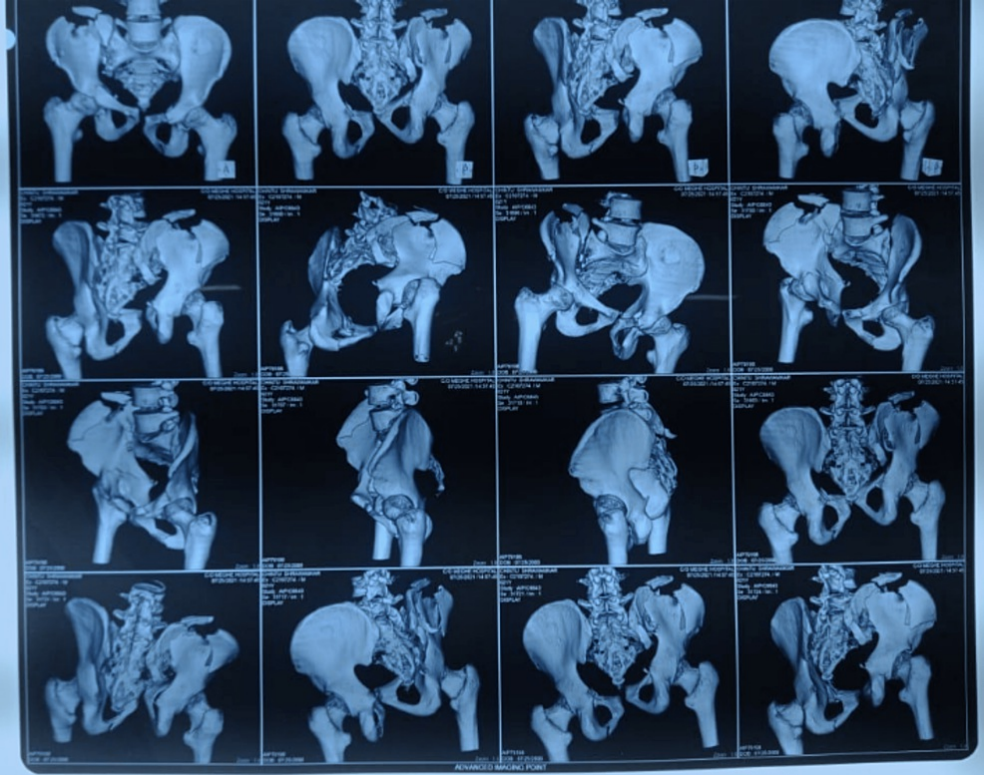
Pre-operative CT images of the patient

Further, inlet and outlet views of the pelvis were done. Further, a CT scan was done. On detailed evaluation, the pelvic fracture was confirmed to be a lateral compression (LC) III injury with a comminuted crescent fracture of the right iliac crest and sacroiliac (SI) joint involvement (Figure 1) with Nakatani type III superior pubic ramus fracture. There was significant soft tissue injury over the site of impact on the right flank region (Figure 1). The patient was informed and consent was acquired for the surgery and other scientific purposes. The pre-operative investigations are shown below in Figures 2, 3.

Operative technique

Once the patient was hemodynamically stabilized, he was planned for definitive fixation. Considering the deep laceration over the right iliac flank, posterior fixation of the crescent fracture was avoided. Keeping the patient in the supine position, close reduction of the crescent crack was attempted with the help of a Schanz pin inserted in either iliac crest and it was used as a joystick. The reduction was checked under fluoroscopy using an inlet, outlet, and AP radiographs. Then an anterior intra-pelvic approach was done and the pubic symphysis diastasis was provisionally reduced with the help of a Farabeuf clamp. The posterior fixation was then completed with the help of two 6.5 mm cannulated cancellous screws placed in the S1 corridor. One long screw was passed engaging the contralateral ilium (120 mm). Anterior fixation was then completed using a 10-holed pre-contoured reconstruction plate, placed bypassing the pubic symphysis fixing the both superior pubic ramus with fractures lateral to the obturator foramen (Nakatani type III). The wound was closed in layers over a drain.

Postoperative imaging

**Figure 4:**
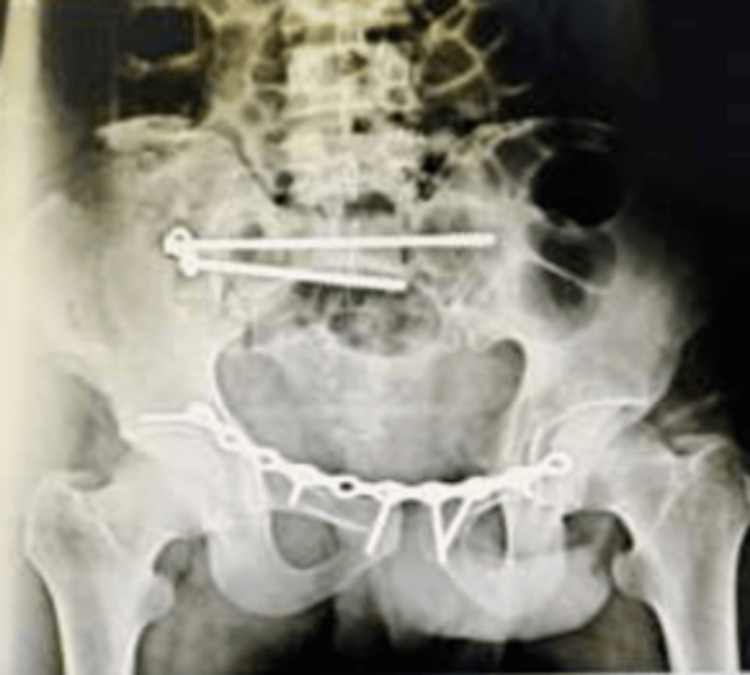
Anteroposterior view of the post-operative X-ray of the patient

**Figure 5:**
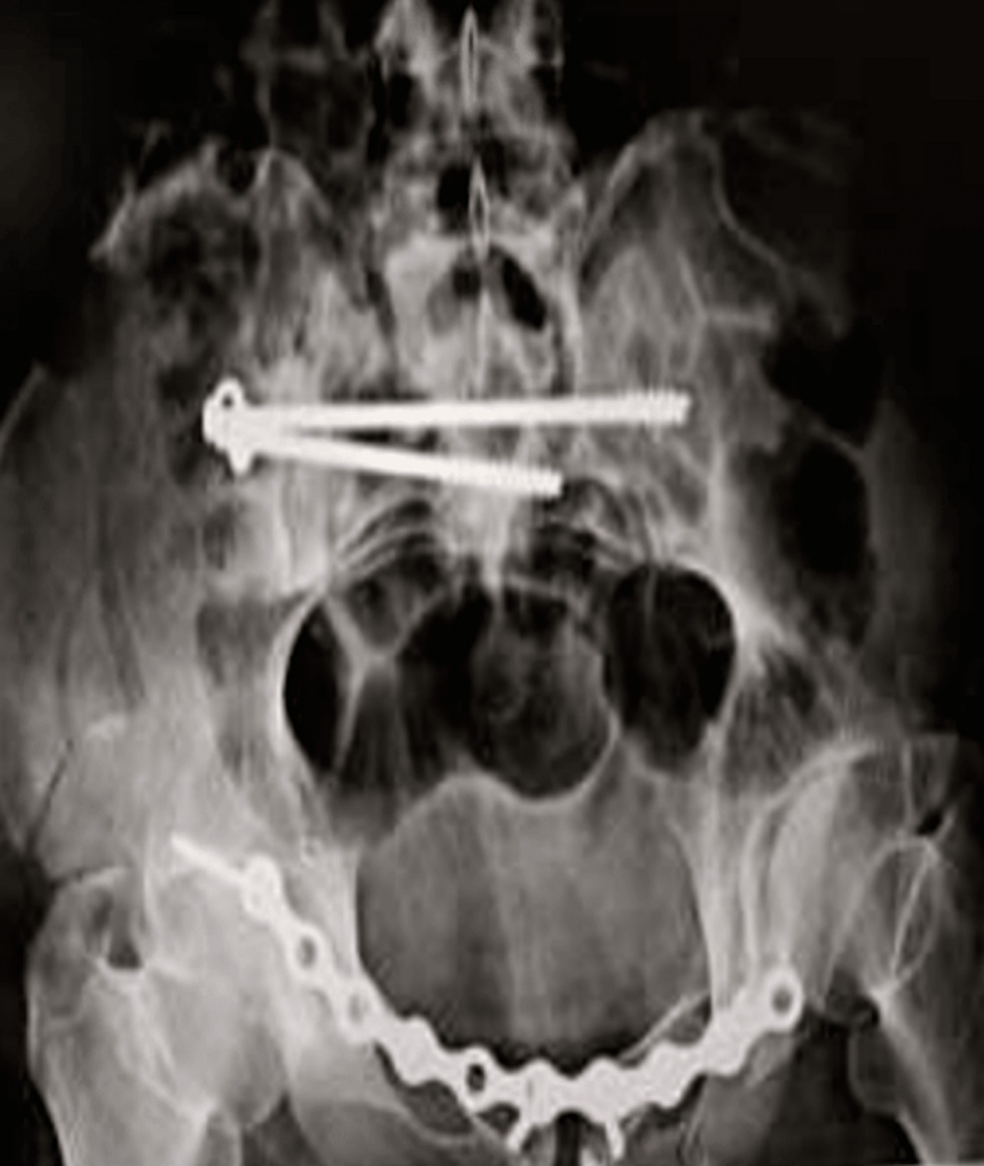
Post-operative X-ray showing pelvic inlet

**Figure 6:**
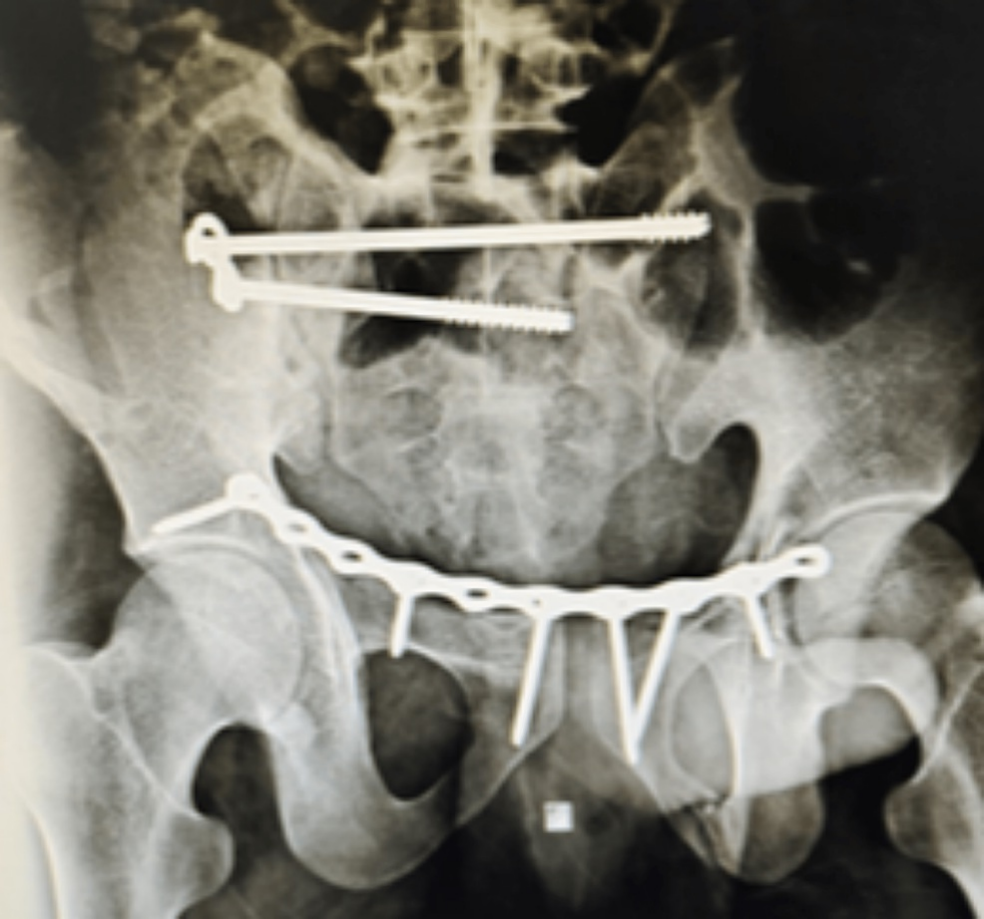
Post-operative X-ray showing pelvic outlet

AP, inlet, and outlet views of the pelvis were done and found to be satisfactory, as shown in Figures 4-6.

Postoperative protocol

A postoperative wound check was done on day two. Deep vein thrombosis (DVT) prophylaxis was given up to 21 days post-surgery. Bedside mobilization was also started after day two. The patient was mobilized non-weight bearing with the help of a walker from the third day. Protected weight bearing was done till eight weeks after surgery. Follow-up X-rays were done after every two weeks till radiological union at three months. Functional outcome was evaluated using Majeed criteria at every follow-up. At the last follow-up of 15 months, the patient could bear weight and perform all activities, including walking, sitting with crossed legs on the floor, and squatting without discomfort. The functional outcome was assessed by the Majeed scoring and came out to be excellent (Majeed score = 90) with no functional limitation at the follow-up.

## Discussion

The surgical treatment of pelvic and acetabular fractures has always required a high level of technical skill. The main objective in mechanically unstable injuries is to achieve precise pelvic ring reduction and anatomical or nearly anatomical restoration of the articulating surfaces. Injury types for isolated pelvic fractures include anteroposterior compression (APC), LC, vertical shear (VS), and mixed injuries. The Tile classification and Young-Burgess classification system have been used to categorize these fracture forms. The older Tile classification has been modified by the Young and Burgess classification. According to this modified classification, pelvic fractures are divided based on the type of force involved as APC, LC, VS, or combined.

The posterior portion of the pelvis is affected by LC damage, which includes a crescent fracture. The incidence rate of crescent fractures is estimated to be between 10% and 15% of all pelvic fractures. According to the Day classification, lateral compression fracture patterns have also been divided into three categories: type I fractures enter the joint inferiorly and involve less than one-third of it, type II fractures involve one-third to two-thirds of it, and type III fractures involve more than two-thirds of it. Type II and type III need to be surgically fixed permanently. Either an open reduction and plating of the anterior SI joint or a closed reduction and percutaneous screw fixation can be used. Open reduction is done using the iliac window and fixation with two perpendicular dynamic compression plates. The benefit of open reduction and internal fixation is that it enables near anatomical reduction, but it requires significant soft tissue dissection with the associated risk of blood loss and postoperative complication in the form of wound dehiscence and increased risk of infection [8].

Crescent fractures can be associated with the Morel-Lavallee (ML) lesion, which is a type of soft tissue injury occurring due to the abrupt separation of skin and subcutaneous tissue with underlying fascia. Concomitant ML lesion is a relative contraindication for ORIF, as it may further increase the risk of postoperative infection. So closed reduction and internal fixation (CRIF) is a preferred modality of posterior ring injury fixation whenever there is a significant soft tissue injury over the gluteal and lumbar region, which is often seen in cases of lateral compression injury. There is a heterogeneity in the nature of crescent fractures. Every time, rotational instability is seen and lateral compression is shown to be the source of injury. Although it is controlled by the sacrospinous and sacrotuberous ligaments, some vertical displacement may happen. It is advised to surgically stabilize crescent fractures to lessen the chance of mal-union and discomfort. Percutaneous fixation of the pelvi-acetabular injury is a demanding procedure considering the close relation with the adjacent neurovascular structures, which require higher precision and has a steep learning curve.

Currently, percutaneous fixation of the pelvi-acetabular injuries is done using intra-operative fluoroscopy, 3D navigation, or with the use of intra-operative CT guidance. Some authors have developed an aiming device for assistance in the placement of screws along the safe corridors in the pelvi-acetabular region. Percutaneous fixation of the SI joint is done using a 6.5 mm partially threaded cannulated cancellous sacroiliac screw or ilio-ileal screw. If there is associated comminution of the sacrum, a fully threaded 7.3 mm cancellous screw can be used to avoid neural foramen compression [9].

In the present case, there was significant soft tissue injury over the right flank with hematoma in the gluteal muscle compartment and internal de-gloving in the left thigh region. So we decided to avoid open reduction and opted for closed reduction and percutaneous screw fixation with SI and ilio-ileal screw placement. The anterior injury was addressed using an anterior intra-pelvic approach with a plate spanning the pubic symphysis [10].

At the recent 15-month follow-up, the patient was walking comfortably with no discomfort and no restriction in the activities of daily living.

## Conclusions

Crescent fracture can have an excellent functional outcome if both the posterior and the associated anterior pelvic ring injuries are addressed with anatomical reduction of the SI joint. Minimally invasive percutaneous fixation of the SI joint is a good modality of fixation with minimal soft tissue injury and can be a viable treatment option if there is an associated ML lesion.
